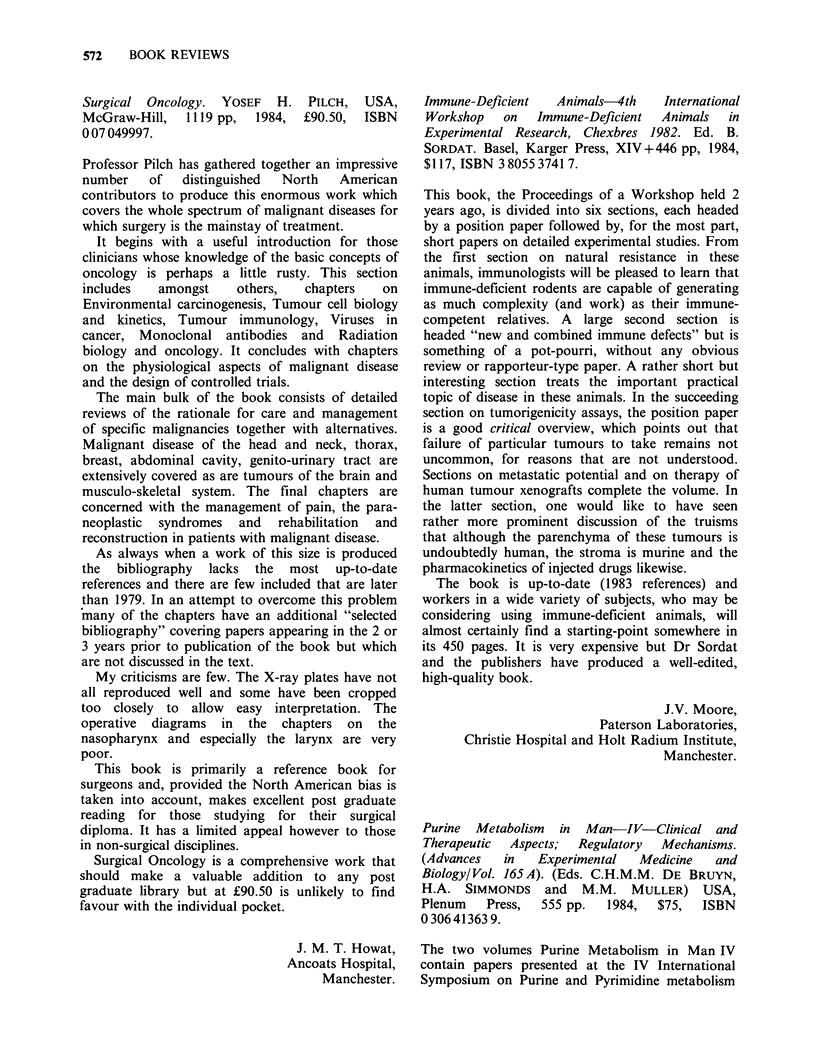# Surgical Oncology

**Published:** 1984-10

**Authors:** J. M. T. Howat


*Surgical Oncology.* YOSEF H. PILCH, USA, McGraw-Hill, 1119 pp, 1984, £90.50, ISBN 0 07 049997.

Professor Pilch has gathered together an impressive number of distinguished North American contributors to produce this enormous work which covers the whole spectrum of malignant diseases for which surgery is the mainstay of treatment.

It begins with a useful introduction for those clinicians whose knowledge of the basic concepts of oncology is perhaps a little rusty. This section includes amongst others, chapters on Environmental carcinogenesis, Tumour cell biology and kinetics, Tumour immunology, Viruses in cancer, Monoclonal antibodies and Radiation biology and oncology. It concludes with chapters on the physiological aspects of malignant disease and the design of controlled trials.

The main bulk of the book consists of detailed reviews of the rationale for care and management of specific malignancies together with alternatives. Malignant disease of the head and neck, thorax, breast, abdominal cavity, genito-urinary tract are extensively covered as are tumours of the brain and musculo-skeletal system. The final chapters are concerned with the management of pain, the paraneoplastic syndromes and rehabilitation and reconstruction in patients with malignant disease.

As always when a work of this size is produced the bibliography lacks the most up-to-date references and there are few included that are later than 1979. In an attempt to overcome this problem many of the chapters have an additional "selected bibliography" covering papers appearing in the 2 or 3 years prior to publication of the book but which are not discussed in the text.

My criticisms are few. The X-ray plates have not all reproduced well and some have been cropped too closely to allow easy interpretation. The operative diagrams in the chapters on the nasopharynx and especially the larynx are very poor.

This book is primarily a reference book for surgeons and, provided the North American bias is taken into account, makes excellent post graduate reading for those studying for their surgical diploma. It has a limited appeal however to those in non-surgical disciplines.

Surgical Oncology is a comprehensive work that should make a valuable addition to any post graduate library but at £90.50 is unlikely to find favour with the individual pocket.

J. M. T. Howat,
Ancoats Hospital,
Manchester.